# Bacterial infection and microbiota in carcinogenesis and tumor development

**DOI:** 10.3389/fcimb.2023.1294082

**Published:** 2023-11-15

**Authors:** Qiao Li

**Affiliations:** Tongji Hospital, Tongji Medical College, Huazhong University of Sciences and Technology, Wuhan, Hubei, China

**Keywords:** microbiota, cancer, tumor microenvironment, sequencing, bacteria

## Abstract

Microbiota colonize exposed body tissues (e.g., gastrointestinal tract, skin, lungs, female genital tract, and urogenital tracts) and unexposed sites (e.g., breast). Persistent bacterial infection in the host lead to the development of multiple disease. They are implicated in the pathogenesis of various complex diseases, including diabetes, atherosclerosis, autoimmune diseases, Alzheimer’s disease, and malignant diseases. Amounting studies have demonstrated the role of bacterial infection in carcinogenesis. The study of microbiota in tumorigenesis is primarily focused on lung cancer, colorectal cancer (CRC), breast cancer, gastric cancer, and gynecologic tumors, and so on. Infection of *Helicobacter pylori* in gastric cancer carcinogenesis is recognized as class I carcinogen by the World Health Organization (WHO) decades ago. The role of *Fusobacterium nucleatum* in the development of colorectal cancer is extensively investigated. Variable bacteria have been cultured from the tumor tissues. The identification of microbiota in multiple tumor tissues reveal that bacterial infection and microbiota are associated with tumor development. The microbiota affects multiple aspects of carcinogenesis and tumor development, including favoring epithelial cells proliferation, establishing inflammatory microenvironment, promoting metastasis, and causing resistance to therapy. On the other hand, microbiota can shape a tumor surveillance environment by enhancing cell activity, and sensitize the tumor cells to immune therapy. In the present review, the roles of microbiota in multiple malignancies are summarized, and unraveling the mechanisms of host-microbiota interactions can contribute to a better understanding of the interaction between microbiota and host cells, also the development of potential anti-tumor therapeutic strategies.

## 1 Introduction

The link between bacterial infection and cancer development was discovered almost a century ago (Chen et al., 2023). It is estimated that 20% of malignancies are related to bacterial infection (Wong-Rolle et al., 2021). However, the role of microbiota in cancer development is not fully elucidated; hence, this topic has attracted considerable research attention worldwide. Identification of microbiota in multiple tumor types were emerging by through high through-put sequencing including 16S rRNA sequencing and Metagenomic sequencing (Fu et al., 2022). The application of bacterial infection in carcinogenesis, tumor metastasis, response to therapy were investigated in gastrointestinal tumors, breast cancer and gynecological tumors (Stein et al., 2000) (Fu et al., 2022) (Wang et al., 2022). Bacteria effect the host directly by malignant transformation of the host cells through secretion of virulence factors or causing inflammatory factors (Odenbreit et al., 2000). Bacteria can also modify the tumor microenvironment by shaping the tumor microenvironment to tumor-promotive or tumor-suppressive directions (Poutahidis and Erdman, 2016) (Dong et al., 2021). The roles of bacterial infection and microbiota in tumor biological functions and various types of tumors will be discussed in different topics in the current review.

## 2 Identification of microbiota in the tumor tissue

**Figure 1:**
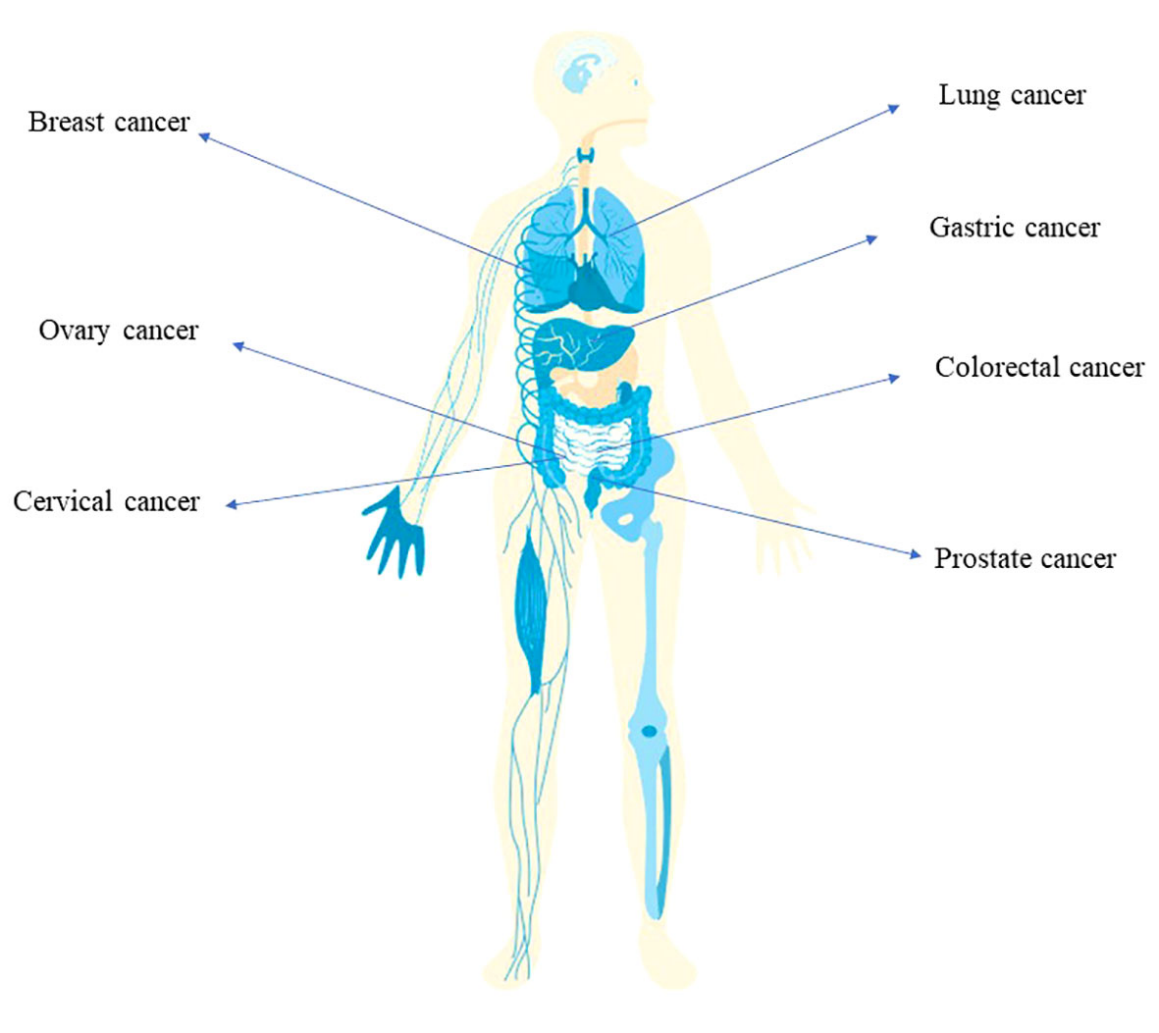
The implication of bacterial infection in multiple cancer types Infection of *Helicobacter pylori* is associated with the development of gastric cancer. The tumorigenesis of colorectal cancer is related with the infection of *E. coli*, *Salmonella* and *Fusobacterium nucleatum*. Also, breast cancer, ovary cancer, lung cancer, prostate cancer involves bacterial infection.

The identification of microorganisms in tumor tissues has been conducted for almost a century (Nejman et al., 2020). Researches have investigated the relationship between microbiota and cancer in multiple solid tumors (Zhao et al., 2021) (Garrett, 2015). By examining the microbiota composition in >1,500 tumor samples (Nejman et al., 2020), Nejman et al. found that bacteria can reside both in macrophages and epithelial cells in tumor tissues (Nejman et al., 2020). Living bacteria can be cultured from tumor samples (Livyatan et al., 2020). The bacterial composition in various types of cancer (e.g., melanoma, ovarian cancer, glioblastoma, pancreatic cancer, breast cancer, and lung cancer) are examined through numerous methods, including 16S-rRNA sequencing, and lipopolysaccharide (LPS) and lipoteichoic acid immunohistochemistry (Nejman et al., 2020). The development of the third-generation sequencing technologies led to the study of the intra-tumoral microbiome (Park et al., 2022). Bacterial 16s ribosomal RNA (16S-rRNA) gene DNA sequencing is a powerful tool for the identification of the bacterial composition in cancer tissues. Costantini et al. identified the bacterial composition in breast cancer through analysis of multi hypervariable 16S-rRNA gene regions (Costantini et al., 2018). The microbiota composition of breast cancer core needle biopsies was analyzed through 16S-rRNA gene sequencing (Costantini et al., 2018). Seven hypervariable regions of the 16S-rRNA gene were simultaneously examined. The results showed that *Proteobacteria* were the most abundant bacteria in the breast cancer tissues (Costantini et al., 2018). *Diaphorobacter*, *Micrococcus*, *Paracoccus*, *Phascolarctobacterium*, and *Ralstonia* are identified to be the most abundant genera in non-small cell lung cancer and the adjacent healthy tissue (Dumont-Leblond et al., 2021). Colorectal cancer (CRC) is colonized by more *Escherichia coli*, *E. faecalis*, *F. nucleatum*, and *Streptococcus gallolyticus* (Chattopadhyay et al., 2021). The identification of bacteria in the tumor tissues gave the direct evidence that microbiota might participate in the development of carcinogenesis (**Figure 1**). But regarding to the role of bacterial infection in tumor tissues as bystander or as effector need further investigation.

Kalaora et al. reported that, apart from antigen presenting cells that can present peptides from bacteria (Pfeifer et al., 1993; Kovacsovics-Bankowski and Rock, 1995; Bettencourt et al., 2020), tumor cells can digest bacteria inside the tumor and present them on tumor cells (Kalaora et al., 2021). Analysis of 17 melanoma metastasis samples extracted from nine patients detected 248 unique human leukocyte antigen class I (HLA-I) and 35 HLA-II peptides in 41 species of bacteria (Kalaora et al., 2021). The presented bacterial peptides can initiate the activation of T-cell response (Kalaora et al., 2021). Moreover, gentamicin protection assay and immunofluorescence staining demonstrated that *Staphylococcus capitis* and *Staphylococcus succinus* from tumors can invade melanoma cells (Kalaora et al., 2021). After co-culture of bacteria with a melanoma-derived melanoma cell line and analysis by HLA peptidomics, HLA-I and HLA-II bacterial peptides were found on cells (Kalaora et al., 2021). This study supported the antigen-presenting function of cancer cells through interaction with microbiota.

## 3 Chronic inflammation induced by bacteria infection in cancer development

The role of bacterial infection in carcinogenesis is complex, and warrant extensive investigation on this topic. All aspects of cancer cells biological behavior can be regulated by microbiota. Bacterial infection can drive the carcinogenesis by multiple mechanisms. Malignant transformation can be induced by bacterial virulence factors. Through producing virulence factor, e.g., *Helicobacter pylori* secret virulence factor which participate in the tumorigenesis of gastric cancer (Kikuchi et al., 2012), *Fusobacterium nucleatum* can produce virulence factors (such as FadA and Fap2) which is implicated in the carcinogenesis of colon cancer (Jawad Abed et al., 2016).

Bacterial infection, especially persistent bacterial infection, cause chronic inflammation which can promote the development of malignancies (Reuter et al., 2010). Bacterial infection can cause the release of reactive oxygen species (ROS) which further activate multiple signaling pathways involving in tumor development, e.g., nuclear factor κB (NF-κB) and signal transducer and activator of transcription 3 (STAT3) (Reuter et al., 2010).

**Figure 2:**
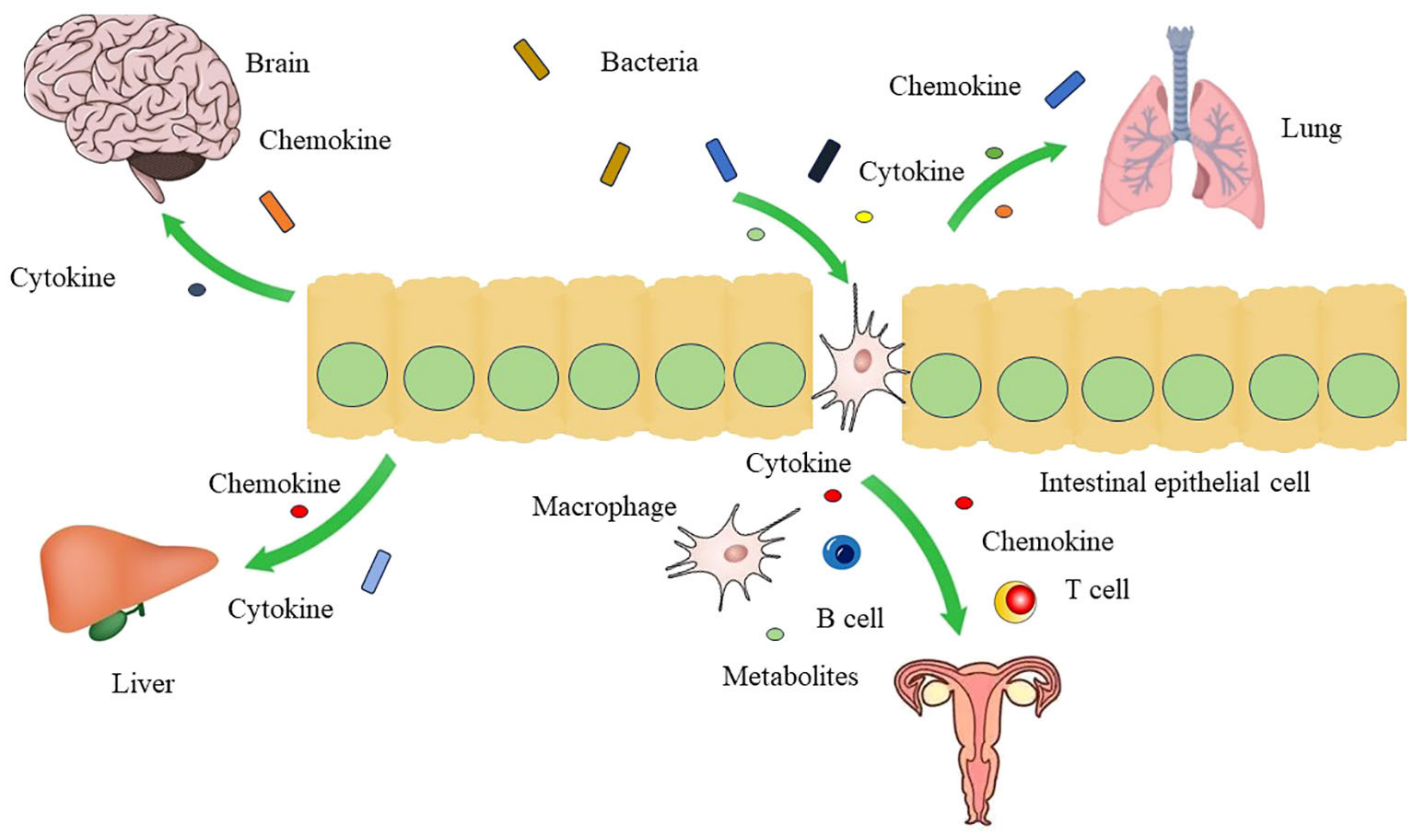
The crosstalk between gut microbiota and other organs Gut microbiota can affect the function of brain, liver, lung and genital tract through gut-brain axis, gut-liver axis, gut-lung axis, and gut-vagina axis by mediation of cytokines, chemokines, and metabolites.

Persistent bacterial infection promotes the malignant transformation of epithelial cells (Normark et al., 2003). Free radicals released by the immune cells, including reactive oxygen species (ROS) and nitrogen oxide species, cause damage to epithelial cells and DNA (Kawanishi et al., 2017). Cytokines and chemokines released by the bacteria facilitate tumor cell growth (Fiorentini et al., 2020) (**Figure 2**). Bacteria remodel the tumor microenvironment (TME) to facilitate its colonization, the presence of bacteria in the TME modulates immune balance to benefit persistent bacterial infections. Bacterial LPS binds to the toll-like receptor 4 (TLR4) receptor of monocytes, thereby shifting their differentiation to the M2 phenotype (Li et al., 2019). Proinflammatory cytokines, including tumor necrosis factor-alpha (TNF-α), IL23, and IL8, can activate immune cells (Hartog et al., 2016). In addition, the accumulation of metabolites and ROS in the tumor microenvironment (TME) can mediate DNA damage and inhibit the function of CD8^+^ T cells (Srinivas et al., 2019).

## 4 Diets effect the microbiota composition and promote cancer development

Diet can affect the gut microbiota composition (Nogal et al., 2021). High fiber and polyphenols, low saturated fats diets will affect the gut microbiota composition (Nogal et al., 2021). High fiber diet increases the microbiota diversity (Nogal et al., 2021). Soluble inulin-type fructans (ITFs), one source of fiber, increase the level of *Bifidobacterium* (Nogal et al., 2021). Mediterranean diet can argument the abundance of *Bifidobacterium*. A ketogenic diet decreases the *Bifidobacterium* levels (Nogal et al., 2021).

Microbiota derived SCFAs enhance regulatory T cells and elicit immune tolerance (Nogal et al., 2021). High fat diet can change the gut microbiota composition by increasing the abundance of *Alistipes*, *Bilophila* and *Bacteroides*, and promote the development of gastrointestinal cancer (Tong et al., 2021).

## 5 Effect of bacterial infection on the tumor microenvironment and tumor metastasis

Infection with viral pathogens can modify the microenvironment of tumor cells. The TME modulated by bacteria enhances cancer development and promotes bacterial infection (Iida N et al., 2013; Poutahidis and Erdman, 2016). Microbiota in the gastrointestinal tract can influence tumor development at remote sites (Zhao et al., 2021), e.g., the composition of gut microbiota modulates lung cancer pathogenesis (Wang et al., 2021). Bacteria from the intestinal lumen translocate to other sites, where they promote tumorigenesis (Wells and Maddaus MA, 1988). The gut microbiota influences the immune environment, for example, *Akkermansia muciniphila* modulates CD8+ T cell response and inhibits colitis-associated tumorigenesis of colon cancer (Wang et al., 2020). Aleksandar et al. reported that *F. nucleatum* modulated the tumor immune microenvironment by recruiting tumor-infiltrating myeloid cells, and promoted tumorigenesis of CRC (Kostic et al., 2013). Bullman et al. reported that *F. nucleatum* was transported with colon cancer cells during metastasis, and facilitated colonization of the metastasis sites (Bullman et al., 2017). Furthermore, whole-genome sequencing of *Fusobacterium* revealed >99.9% average nucleotide identity between the primary and metastatic tumors (Bullman et al., 2017). Fu et al. reported that tumor-resident intracellular microbiota promoted the metastasis of breast cancer cells (Fu et al., 2022); live bacteria, including *Staphylococcus*, *Lactobacillus*, *Enterococcus*, and *Streptococcus*, can be cultured from breast cancer tissues (Fu et al., 2022). The investigators traced the motility of the bacteria *in vivo* through fluorescent labeling (Fu et al., 2022). They found that bacteria were transported along with tumor cells in the circulation; intracellular bacteria facilitated the establishment of metastatic colonization sites by tumor cells (Fu et al., 2022).

## 6 Role of microbiota in the regulation of response to therapy

Intratumor bacteria not only affect the metastasis of cancer cells, but also affiliate resistance to therapy of cancer cells. The microbiota in tumors modify the immune response in the TME, and regulate the response to anti-tumor therapy. Bacterial infection can shift the immune response (Kim and Covington A, 2017) against tumor cells to a tumor-permissive state. The composition of the gut microbiota can affect the response of cancer cells to immune checkpoint blockade immunotherapy (Gopalakrishnan et al., 2018). Gopalakrishnan et al. reported that, in patients with melanoma who received anti-PD-1 immunotherapy, gut microbiota varied between the responders and non-responders (Gopalakrishnan et al., 2018). Higher alpha diversity in the composition of fecal microbiota was observed in responders versus non-responders (Gopalakrishnan et al., 2018). The gut microbiome can affect the efficacy of PD-1-based immunotherapy in multiple tumor types (Long et al., 2019). Disturbance of the gut microbiome composition can increase the resistance to immune checkpoint inhibitors that target the PD-1/PD-L1 axis (Routy et al., 2018). Transplantation of fecal microbiota from responders to germ-free mice increases the therapeutic efficacy of PD-1 blockade (Routy et al., 2018). In contrast, transplantation of fecal microbiota from non-responders to germ-free mice did not ameliorate the response to therapy (Routy et al., 2018). A correlation between the abundance of *Akkermansia muciniphila* and response to immune checkpoint inhibitor therapy exists, as shown by a metagenomics analysis of stool samples obtained from patients (Routy et al., 2018). In colon cancer, *Fusobacterium* mediates chemoresistance to capecitabine (and 5-FU) by TLR4/MyD88-driven activation of autophagy (Yu et al., 2017). *F. nucleatum* infection induces light chain 3-II (LC3-II) expression, increases the RNA and protein expression of autophagy-related proteins, such as, unc-51 like autophagy activating kinase 1 (ULK1), phosphorylated-ULK1, and autophagy related 7 (ATG7) (Yu et al., 2017). Antibiotic treatment can reduce the tumor load of the colorectal cancer and may benefit the treatment of colorectal cancer positive with *Fusobacterium* (Bullman et al., 2017).

## 7 Role of microbiota in gastric cancer

Gastric cancer is the fifth most common type of cancer worldwide, and is associated with a high incidence rate in Asian countries (Smyth et al., 2020). Gastric cancer can be divided to 2 histological types: intestinal-type and diffuse-type carcinomas (Yasui et al., 2011). Pathologically, approximately 90% of gastric cancers are adenocarcinomas. The development of gastric cancer involves a series of pathological steps (i.e., Correa’s steps), including atrophy, intestinal metaplasia, and dysplasia to adenocarcinoma (Sheh A, 2013). Compared with other body sites, the colonization of the stomach is challenging (Sheh A, 2013). The low pH 1–2 in the stomach is apparently formidable and hospitable for bacterial colonization (Sheh A, 2013). This contributes to significant differences in microbiota composition between the stomach and other sites of the gastrointestinal tract (Stearns et al., 2011). The cascade of events underlying the process from gastritis to intestinal metaplasia and gastric adenocarcinoma may require decades. Notably, the colonization by microbiota changes during this process (Alarcón and Llorca L, 2017).

**Figure 3:**
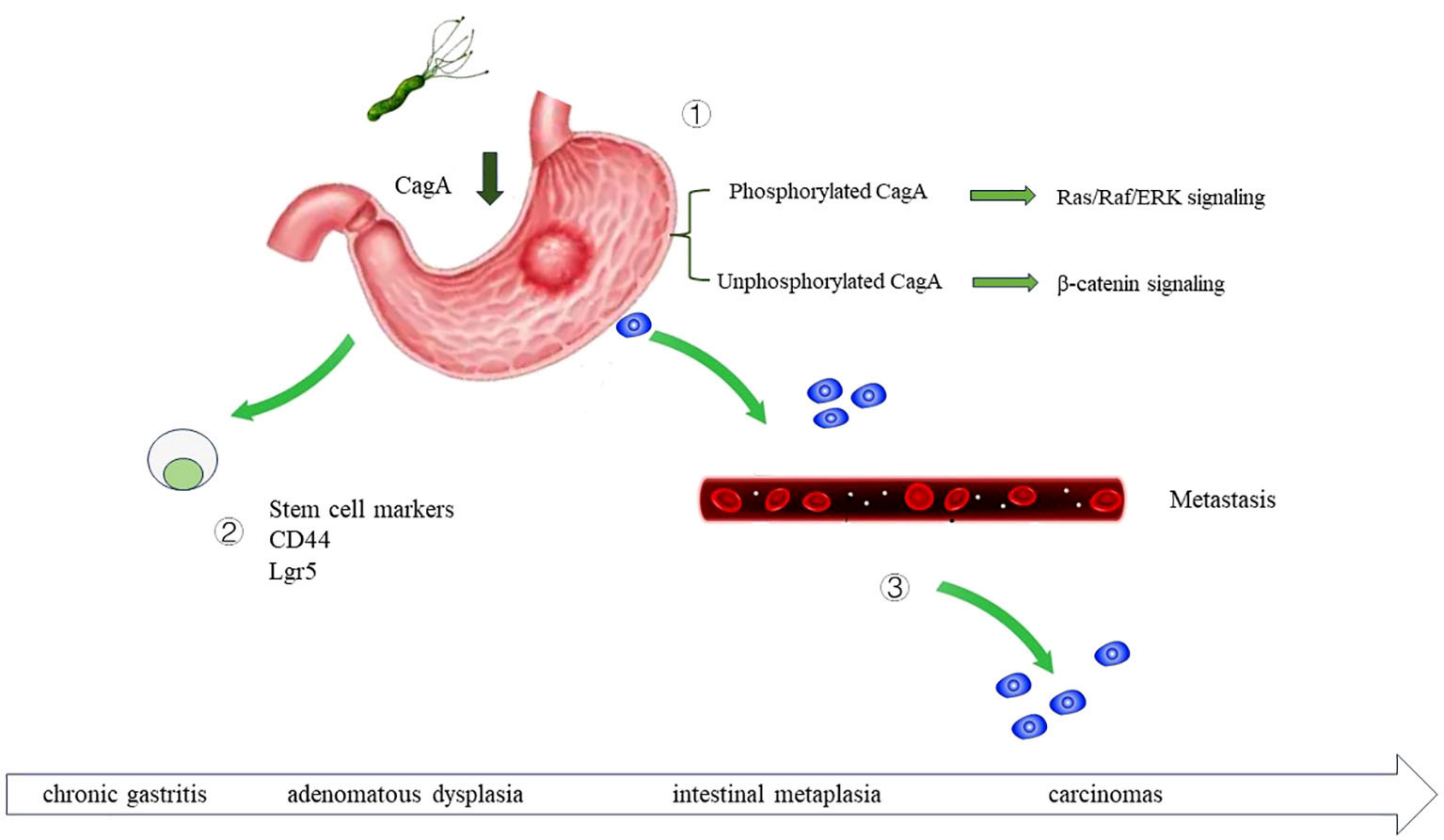
The role of *H. pylor*i infection in gastric cancer carcinogenesis *H. pylori* infection can drive the development of gastric cancer through multiple mechanisms. By secreting virulence factor CagA, upregulating the stem cell traits of gastric epithelial cells, and promote the metastasis of gastric cancer cell. ① Once injected into the host cells, CagA is phosphorylated at glutamate-proline-isoleucine-tyrosine alanine (EPIYA) motifs. Phosphorylated CagA activate Ras/Raf/ERK signaling, and further activate the transcription of cyclin D and promote cell proliferation. Non-phosphorylated CagA activate β-catenin signaling and further upregulate cyclin D1 and c-Myc. ② *H. pylori* regulate the stem cell traits of gastric epithelial cell, increase CD44, Sox2, Oct4 and Nanog expression. ③ *H. pylori* can accelerate the metastasis of gastric cancer cell.

The relationship of bacterial infection with tumorigenesis has been recognized since the discovery of *H. pylori* decades ago (Wang et al., 2014). *Helicobacter pylori* (*H. pylori*) infection in Mongolian gerbil is an established model for identifying the role of bacterial infection in the carcinogenesis and development of gastric cancer (Wang et al., 2014). *H. pylori* infection promotes the development of gastric cancer through multiple steps. But only 1–3% of the population infected by *H. pylori* will develop gastric cancer (Wang et al., 2014). Cag pathogenicity island (Cag PAI) of the bacteria is a key determinant in the malignant transformation of gastric epithelial cells by *H. pylori* (Odenbreit et al., 2000; Kikuchi et al., 2012). Virulence factors of the bacteria Cag PAI can be injected into the cytosol of gastric epithelial cells through the syringe-like structure type IV secretion system (Odenbreit et al., 2000).Subsequently, the tyrosine residue on four distinct glutamate-proline−isoleucine−tyrosine−alanine (EPIYA) motifs at the C-terminal region of CagA is phosphorylated (Stein et al., 2000). CagA promotes the acquisition of a ‘hummingbird’ phenotype by epithelial cells and the epithelial–mesenchymal transition of epithelial cells (Baj et al., 2020), thereby promoting the migration of cells (Kikuchi et al., 2012; Zhang et al., 2022). And increases the stem cell traits of gastric epithelial cells. Moreover, it increases the expression of gastric cancer stem cell marker CD44 (Bessède et al., 2014). CagA of *H. pylori* activates the Wnt signaling of the gastric epithelial cells, and drives carcinogenesis (Guo et al., 2022) as shown in **Figure 3**. Apart from the effect of the bacterial virulence factor, persistent inflammation induced by chronic *H. pylori* infection can accelerate the development of gastric cancer (Normark et al., 2003). Eliminating *H. pylori* infection by antibiotics proved to be beneficial to the patients (Wong et al., 2004). After 7.5 years’ follow-up, *H. pylori* eradiation in precancerous lesions group including gastric atrophy, intestinal metaplasia, and dysplasia group, prevent the gastric cancer carcinogenesis (Eun et al., 2014).

Except for *H. pylori*, other bacteria also reside in the stomach. By pyrosequencing methods, Eun et al. profiled the bacteria composition in the patients with chronic gastritis, intestinal metaplasia, and gastric cancer (Eun et al., 2014). Significantly different microbiota composition was identified in the gastric mucosa from *Helicobacter* colonized gastric cancer patients compared to gastritis and intestinal metaplasia patients (Eun et al., 2014). Through sequencing analysis, Dai et al. detected significant differences between gastric cancer tissues and matched non-tumor tissues (Dai et al., 2021).

The composition of the microbiota in gastric cancer were reported by different studies (Sheh A, 2013) (Bik et al., 2006) (Stewart and Wu F, 2020). It has been demonstrated that *Firmicutes*, *Proteobacteria*, and *Bacteroidetes* are the most abundant phyla in the microbiota in the stomach (Sheh A, 2013). Consistent results were reported by Elisabeth et al. that *Proteobacteria*, *Firmicutes*, *Bacteroidetes*, *Actinobacteria*, and *Fusobacteria* phyla were the dominant bacteria in the stomach (Bik et al., 2006) (Stewart and Wu F, 2020). Ferreira et al. determined the microbiota composition of 54 patients with gastric carcinoma and 81 patients with chronic gastritis, through 16S rRNA gene next-generation sequencing (Ferreira et al., 2018). And it was found that a reduced microbial diversity was present in the gastric carcinoma microbiota (Ferreira et al., 2018). *Proteobacteria*, *Firmicutes*, *Bacteroidetes*, *Actinobacteria* and *Fusobacteria* were the most dominant bacteria in the stomach. And this is consistent with other studies (Bik et al., 2006) (Stewart and Wu F, 2020). Elucidating the role of microbiota in the gastric cancer will introduce a new dimension of the microbiome in the common malignancies and benefit the development of therapeutic strategies.

## 8 Role of microbiota in colorectal cancer

CRC is the third leading cause of cancer-related death worldwide (Siegel et al., 2022 2022). CRC is related to mutation of the adenomatous polyposis coli (APC) tumor suppressor gene, which activates the Wnt/β-catenin signaling pathway (Bian et al., 2020; JvP et al., 2021; Zhao et al., 2022). The translocation of β-catenin into the nucleus can activate the signaling pathways related to the development of cancer (Bian et al., 2020). The involvement of bacterial infection in colon cancer has been reported for decades. It has been shown that an imbalance in colon microbiota was tumorigenic. In addition, the occurrence of colon cancer has been linked to dietary habits (i.e., consumption of red meat, animal fat, and alcohol) (Campos et al., 2005). Notably, smoking has been associated with a high incidence of CRC (Bai et al., 2022).

Diet composition can affect the production of short-chain fatty acids (SCFAs), including acetate, propionate, and butyrate, which regulate the function of intestinal epithelial cells (Louis and Hold GL, 2014). Among them, butyrate and propionate inhibit histone deacetylases (HDACs) and regulate the function of CD8+ T cells (Bai et al., 2022) (Louis and Hold GL, 2014). The level of propionate in colorectal cancer decreases. Propionate regulate the mTORC2/PDK1/AKT signaling pathway (Louis and Hold GL, 2014). The metabolites from the microbiota modify the biological behaviors of the cancer cell. SCFAs propionate and butyrate, by-products of the intestinal bacterial fermentation, can induce autophagy of colon cancer cell and retard the cellular apoptosis due to mitochondrial dysfunction (Tang et al., 2011). Increased LC3-II and reduced p62/SQSTM1 were observed in the propionate treated colon cancer cells (Tang et al., 2011). It also indicated the therapeutic potential of SCFAs might be enhanced by autophagy inhibitor (Tang et al., 2011).

It is estimated that a load of 10^10^ to 10^12^ CFU/g bacteria is contained in the gastrointestinal tract (Korecka A, 2012; Bretin and Gewirtz AT, 2018). After sequencing nine metastatic CRC tissues and seven non-metastatic CRC tissues (Chen et al., 2020), Chen et al. found that *Fusobacteriaceae* is more frequently present in patients with metastatic CRC versus those with non-metastatic CRC (Chen et al., 2020). The association of *Escherichia coli* (*E. coli*) with colon cancer was reported in 1998 (Swidsinski et al., 1998). Based on quantitative polymerase chain reaction and 16S-rRNA sequencing analyses, Swidsinski et al. identified intracellular *E. coli* in mucous obtained from patients with colorectal carcinoma (Swidsinski et al., 1998). In addition, Long et al. reported that *Peptostreptococcus anaerobius* promoted carcinogenesis of CRC (Long et al., 2019).

The composition of colorectal cancer microbiota was investigated by different groups. *Fusobacterium*, *Peptostreptococcus*, *Porphyromonas*, *Bacteroides*, *Parvimonas*, *Prevotella*, *Gemella*, *Streptococcus, Clostridium*, *Escherichia*, *Bilophila*, *Campylobacter*, *Phascolarctobacterium*, *Selenomonas*, *Ruminococcus*, *Shigella*, *Akkermansia*, *Desulfovibrio*, *Eubacterium*, *Leptotrichia* are among the most common bacteria in colorectal cancer (Ternes et al., 2020).Zhou et al. identified “*Bifidobacteria*, *Fusobacterium nucleatum*, *Geotrichum candidum*, *Porphyromonas asaccharolytica*, *Escherichia coli*, *Rhodococcus*, *Anaerostipes caccae*, *Enhydrobacter*, *Lachnoclostridiumsp*.*m3*, *Bacteroides clarus*, *Clostridium hathewayi*, *Ruminococcaceae*, *Bacteroides thetaiotaomicron*, *Culinariside*, and enterotoxigenic *Bacteroides fragilis* (ETBF)” as biomarkers in gut microbiome as early diagnostic markers of colorectal cancer (Zhou et al., 2022). *Fusobacterium nucleatum*, *Escherichia coli*, and *Bacteroides fragilis* are among the most evaluated bacteria in the carcinogenesis of colorectal cancer (Zhou et al., 2022). Experimental evidence supporting the role of *Fusobacterium nucleatum*, *Escherichia coli*, and *Bacteroides fragilis* in colorectal cancers substantially increased (Tilg et al., 2018).

### 8.1 The relationship of *Fusobacterium nucleatum* infection with colon cancer

The relationship between *F. nucleatum* and CRC is well established (Bashir et al., 2015). *F. nucleatum* is an opportunistic, Gram-negative bacterium that has been associated with the metastasis of colon cancer cells. *F. nucleatum* reaches the gastrointestinal tract through the oral cavity or by hematogenous translocation (Ashare et al., 2009; Kostic et al., 2013). Bacterial infection modifies the phenotype of macrophage to tumor promoting phenotype (Chen et al., 2018). Monocytes differentiate toward the M1 and M2 phenotypes under different stimuli. M2 macrophages, termed alternatively activated macrophages, promote tumor development and cancer cell metastasis, and inhibit anti-tumor immunity (Mantovani et al., 2002). *In vitro*, *F. nucleatum* infection favors the polarization of macrophages toward the M2 phenotype. This process depends on the signaling of pattern-recognition receptor TLR4. It modulates the polarization of macrophages toward the CD206^+^ M2 phenotype in the TME, and promotes the development of colorectal tumors in a TLR4-dependent mechanism (Chen et al., 2018). Typically, the activation of LPS-TLR4 signaling lead to the polarization of macrophages toward the M1 phenotype. However, evidence revealed that bacterial infection induced the differentiation of macrophages to the M2 phenotype by IL6/STAT3/c−MYC signaling (Chen et al., 2018).

**Figure 4:**
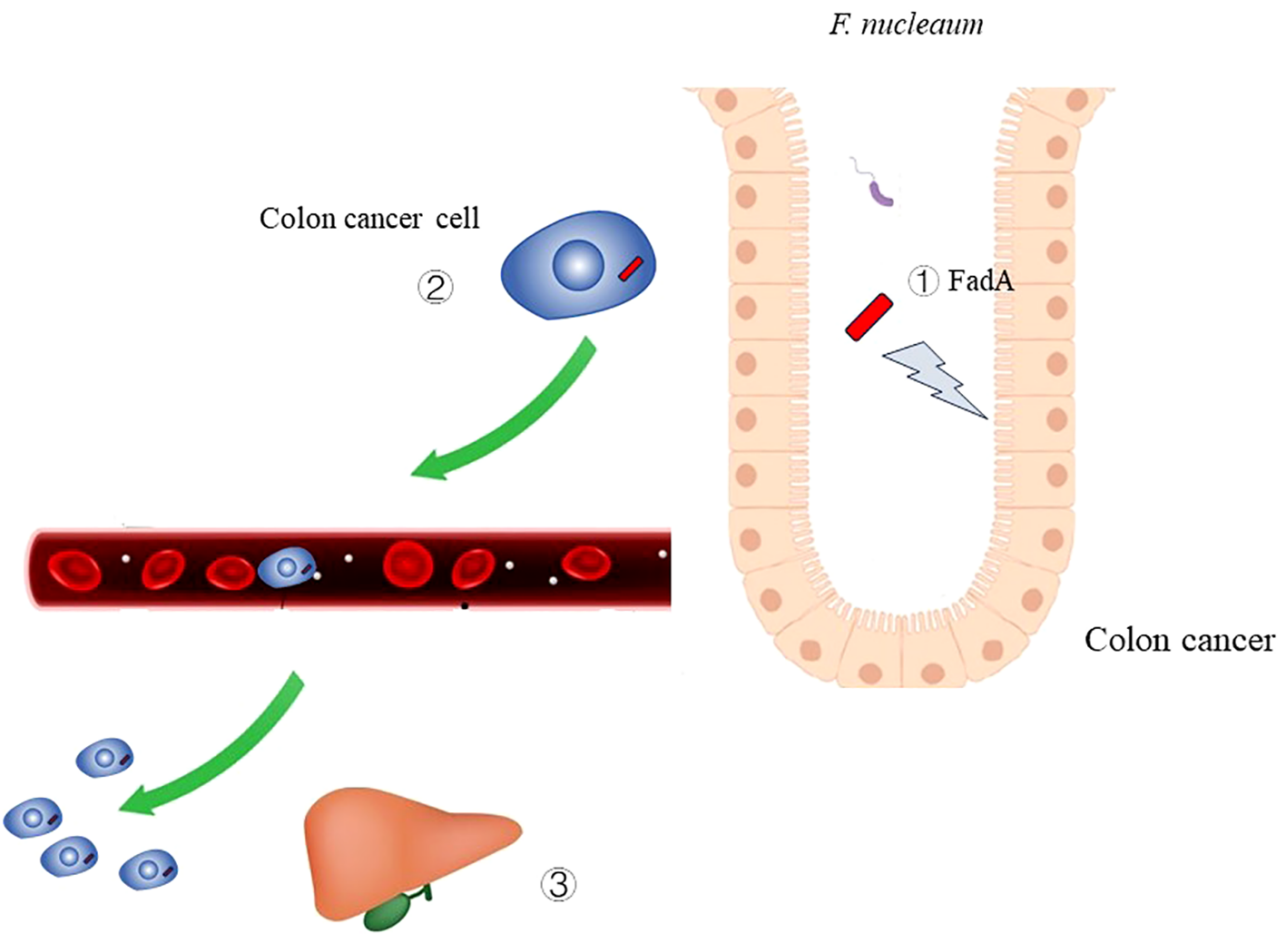
The role of *Fusobacterium nucleatum* infection in colon cancer ① Virulence factor FadA of *F. nucleaum* can cause damage to DNA.② *F. nucleaum* can metastasize along with colon cancer cell.

The migration and invasion of colorectal cancer can be regulated by gastrointestinal microbiota (JvP et al., 2021). *Fusobacterium nucleatum* drive colorectal cancer migration by inducing IL-8 and cytokine CXCL1 secretion (Casasanta et al., 2020). Han et al. reported that *F. nucleatum* promoted liver metastasis of CRC (Yin et al., 2022) as shown in **Figure 4**. Following infection of mice with *F. nucleatum* through the oral route, metastasis of CRC cells to the liver was increased, whereas the body weight and overall survival time of the mice were decreased (Yin et al., 2022). The bacterium modified the TME of the liver by recruiting Th17 cells and regulatory T cells (Yin et al., 2022). Furthermore, *F. nucleatum* binds to the inhibitory immune receptor targeting the T cell immunoglobulin and ITIM domain (TIGIT) of immune cells through Fap2 (Garrett, 2019). This leads to inhibition of the function of tumor-infiltrating lymphocytes and natural killer cells, thereby facilitating the immune evasion of tumor cells (Garrett, 2019).

Activation of NF-κB signal mediated by Toll-like receptors and nucleotide-binding oligomerization domain-like receptors bind with *Fusobacterium nucleatum*, induce chronic inflammation (Garrett, 2015). Activation of IL-6, TNF and STAT3 signaling is also tumor promoting (Garrett, 2015).

### 8.2 The relationship of *E. coli* infection with colon cancer

Bacterial infection can lead to DNA damage and mutation caused by the metabolites of the bacteria. *E. coli* harboring the polyketide-nonribosomal peptide synthase operon (pks) island produces colibactin, thereby causing genomic instability of the host cell (Nougayrede et al., 2006). Colibactin secreted by *Escherichia coli* activates the senescence-associated secretory phenotype and promotes the development of colon cancer (Dalmasso et al., 2014). *E. coli* harboring the pks island causes DNA double-strand breaks. Pleguezuelos-Manzano et al. analyzed 5,876 human cancer genomes and reported that *pks+ E. coli* induced a distinct mutational signature in 20% of the healthy population, 40% of patients with inflammatory bowel disease, and 60% of patients with CRC (Pleguezuelos-Manzano et al., 2020).

Enterotoxigenic *Bacteroides fragilis* (ETBF) has been found in biofilms extracted from the colon of patients with familial adenomatous polyposis (Dejea et al., 2018). This bacterium secretes a metalloprotease toxin (*Bacteroides fragilis* toxin). The occurrence of familial adenomatous polyposis is related to mutation in the APC tumor suppressor gene. ETBF in CRC can cleave and degrade E-cadherin through the secretion of metalloproteases (Dejea et al., 2018). *E. coli* and ETBF are dominant bacteria in the biofilm of patients with CRC. Wu et al. reported that ETBF promoted colon cancer tumorigenesis by activating T helper type 17 (Th17) T cell responses (Wu et al., 2009). Use of antibodies against IL17 and IL23 receptor alleviated the inflammatory infiltration and hyperproliferation of colonic mucosal cells (Wu et al., 2009).

### 8.3 The relationship of *Salmonella* species infection with colon cancer

*Salmonella* species (Gram-negative bacteria) can cause various diseases, ranging from self-limiting gastroenteritis to typhoid fever (Bretin and Gewirtz AT, 2018). Enteric *Salmonella* infection*, e*.g., *Salmonella* Enteritidis, has been associated with CRC (Mughini-Gras et al., 2018). *Salmonella* infection in the azoxymethane/dextran sodium sulfate mouse model or APC-deficient mice model significantly increased the incidence of CRC (Lu et al., 2014). Moreover, this relationship involves the *Salmonella* AvrA protein, which is expressed at higher levels in tumor-adjacent versus non-cancer colorectal mucosa (Lu et al., 2014; Lu et al., 2016; Lu et al., 2017). AvrA activates the STAT3 and β-catenin signaling pathways (Lu et al., 2014; Lu et al., 2016).

## 9 Role of microbiota in gynecologic tumors

Female genital tract is occupied by microbiota. Gynecologic cancer refers to a group of cancers of the female reproductive organs (e.g., cervical, endometrial, and ovarian). The association between bacterial infection and gynecologic tumors is complex. Due to its anatomy, the female genital tract is exposed to the external environment. This provides an opportunity for bacteria to enter the reproductive organs. *Lactobacillus* predominantly colonizes low-grade squamous intra-epithelial lesions compared with invasive cervical cancers (Mitra et al., 2015). Greater diversity in vaginal microbiota correlates with more severe cervical intra-epithelial neoplasia disease (Mitra et al., 2015). This finding indicated that *Lactobacillus* exerts protective effects against cervical cancer tumorigenesis, possibly by promoting human papillomavirus clearance (Mitra et al., 2015). Persistent infection with *Neisseria gonorrhoeae* is associated with urethritis (Unemo et al., 2019). *Neisseria gonorrhoeae* has been associated with malignant transformation due to the regulation of cyclin B (CCNB) expression and the induction of cell cycle arrest in the G1 phase (Jones et al., 2007). The intratumoral abundance of *F. nucleatum* may function as a prognostic marker for cervical carcinoma (Huang et al., 2020).

The composition of gynecologic tumors was elucidated by multiple researches. High microbial diversity and overgrowth of anaerobic bacteria were found in the vaginal microbiome of cervical cancer (Laniewski et al., 2020). *Atopobium* and *Porphyromonas* are associated with endometrial cancer (Laniewski et al., 2020). *Brucella*, *Mycoplasma* and *Chlamydia*, *Acinetobacter* are related with ovary cancer (Laniewski et al., 2020). Dysbiosis of female reproductive tract (FRT) are emerging as one of the drivers of gynaecological malignancies (Laniewski et al., 2020).

Ovarian cancer is the most common type of gynecologic cancer, and is typically diagnosed at the late stage of the disease (Lengyel, 2010). Bacteria can also colonize the ovary through the pelvic cavity, particularly in patients suffering from chronic pelvic inflammatory disease (Infectious Vaginitis, 2023). Studies reported that microbiota play multiple functions in gynecologic tumors (Routy et al., 2018). For example, the abundance of *Akkermansia* correlated with the response of patients to immunotherapy with immune checkpoint inhibitors targeting programmed cell death 1 (PD-1) (Routy et al., 2018). Studies analyzed ovarian cancer samples through 16S-rRNA sequencing, using distal fallopian tube samples as control (Zhou et al., 2019). The results demonstrated that *Proteobacteria* and *Firmicutes* are the predominant phyla in ovarian cancer by Lefse (LDA Effect Size) analysis (Zhou et al., 2019). Genera within *Firmicutes* can produce butyrate which is tumor preventive in the early stages of tumor development (Zhou et al., 2019). Higher abundance of *F. nucleatum* correlates with lower overall survival and progression-free survival rates, as well as enhanced characteristics of cancer stem cells (Huang et al., 2020). Wang et al. reported that transplantation of fecal microbiota from patients with ovarian cancer to a mouse model of ovarian cancer accelerated tumor development. Interestingly, *Akkermansia* supplementation in mice reversed this effect (Wang et al., 2022).

The female genital tract is adjacent to the urinary tract (Walther-António et al., 2016). Walther-António et al. found that the uterine microbiome contributed to the development of endometrial cancer (Walther-António et al., 2016). Through microbiome sequencing (16S-rDNA V3–V5 region), researchers identified the bacterial composition in endometrial cancer (Walther-António et al., 2016). It is found that *A. vaginae* and *Porphyromonas* sp. correlate with presence of endometrial cancer (Walther-António et al., 2016). This study proposed the potential roles of microbiota in the progression of endometrial cancer (Walther-António et al., 2016). Hawkins et al. reported that *Actinobacteria*, *Bacteroidetes*, *Firmicutes*, OD1, and *Proteobacteria phyla* were present in both benign and malignant uterine tissue specimens (Mitra et al., 2015). Comparison of the microbial profiles (at the genus level) revealed that the microbial diversity is greater in endometrial cancer versus the benign uterus (Hawkins et al., 2022).

## 10 Role of microbiota in urinary carcinomas

Bladder cancer is the tenth most common type of cancer worldwide (Bray et al., 2018), and the predominant tumor type of the urological tract. Several research groups have discovered that the urinary tract is colonized by unique urinary microbiota (Parra-Grande et al., 2022) (Martin et al., 2022). In urinary carcinomas, the microbiome promotes the carcinogenesis of epithelial cells through different mechanisms (Parra-Grande et al., 2022). Persistent bacterial infection can induce DNA mutation and genomic instability (Hanahan and Weinberg, 2011; Hanahan, 2022). Moreover, ROS and metabolites, such as short-chain fatty acids, can modify the urinary microenvironment and promote tumorigenesis (Hanahan and Weinberg, 2011; Hanahan, 2022). In addition, bacteria (e.g., *E. coli*) can persistently survive in bladder epithelial cells (Hannan et al., 2012).

The composition of the urinary carcinomas’ microbiota was verified. In bladder cancer patients’ samples, abundant levels of *Proteobacteria*, *Firmicutes* and *Actinobacteria* at the phylum level are more common in the urinary microbiota of bladder cancer patients than in control patient (Karam et al., 2022). *Fusobacterium*, *Acinetobacter*, *Cupriavidus*, *Corynebacterium*, *Streptococcus* and *Staphylococcus* are higher in bladder cancer urine samples (Karam et al., 2022). In tissue samples, *Firmicutes*, *Bacteroidetes*, *Proteobacteria* and *Actinobacteria* were detected on the phyla level both in bladder cancer patients and normal samples (Karam et al., 2022). With lower abundance of *Firmicutes* and *Bacteroidetes*, higher abundance of *Proteobacteria* and *Actinobacteria* presented in bladder cancer samples (Karam et al., 2022). *Streptococcus* have a prominent abundance in the urine microbiota of prostate cancer patients (Alanee et al., 2019) (Karam et al., 2022).

Apart from 16S-rRNA sequencing, shotgun metagenomic sequencing and microbiome metabolomics have been used to identify the microbiota composition in bladder cancer (Zhang et al., 2023). Parra-Grande et al. compared the microbial composition between matched tumor and non-tumor samples (Parra-Grande et al., 2022). They observed lower biodiversity in the tumor samples than in the mucosa of healthy controls (Parra-Grande et al., 2022). The most abundant phyla in the tumor and non-tumor samples were *Firmicutes*, *Bacteroidetes*, *Proteobacteria*, and *Actinobacteria* (Parra-Grande et al., 2022). Higher abundance of *Actinobacteria* was observed in the non-tumor mucosa samples versus the tumor samples (Parra-Grande et al., 2022). Notably, higher abundance of *Enterococcus* correlated with lower tumor grade (Parra-Grande et al., 2022).

## 11 Role of microbiota in lung cancer

Lung cancer is the most common type of cancer worldwide (Avasarala and Rickman, 2022). Small cell lung cancer and non-small cell lung cancer are the most common categories of lung cancer. The latter accounts for up to 85% of lung cancer cases. The most common types of lung cancer include adenocarcinoma, squamous cell carcinoma, and large cell carcinoma (Jackson et al., 2001).

The role of the microbiome in lung cancer has been more and more recognized by researchers (Jin et al., 2019) (Zhao et al., 2021). Bacteria can colonize the lungs and induce chronic inflammation, while various cytokines and chemokines can facilitate tumor growth. Not only the lung microbiota is involved in the carcinogenesis of lung cancer (Jin et al., 2019), but also the microbiome in the intestinal lumen can modify the TME of lung cancer from remote sites (Budden et al., 2017). Lung cancer carcinogenesis is affected by the gut microbiota (Liu et al., 2021). The crosstalk between the lungs and intestines is involved in the development of lung cancer (Budden et al., 2017) (He Zhuang et al., 2019). It has been shown that the transportation of Th17 cells from the intestines to the lungs aggregated immune imbalance in the lungs (Liu et al., 2021). Zheng et al. reported that patients with early-stage lung cancer have a specific gut microbial profile (Zheng et al., 2020). Different microbial compositions in the gut correlate with the stages and subtypes of lung cancer, indicating that a gut microbiota signature may predict the development of lung cancer (Zheng et al., 2020).

The composition of the microbiota in lung cancer were not extensively reported as gastrointestinal cancer (Xu et al., 2020). Increase of oral taxa *Streptococcus* and *Veillonella* was detected in the lower airways of lung cancer patients (Pizzo et al., 2022). It is related with ERK and PI3K signaling pathway activation (Pizzo et al., 2022). Enrichment of oral bacteria *Prevotella*, *Veillonella*, *Rothia*, *Streptococcus*, and *Porphyromonas* in the lower airway were reported by multiple studies (Wu et al., 2017). Lower alpha diversity of bacterial community was reported in lung cancer patients than in non-malignant lung tissues (Pizzo et al., 2022). *Herbaspirillum* and *Sphingomonadaceae* are more common in lung cancer tissues than normal lung tissues (Jin et al., 2019). Enrichment of *Firmicutes*, *Granulicatella*, *Abiotrophia*, and *Streptococcus* with a decreased bacterial community diversity were reported in lung cancer patients (Lee et al., 2016). Enrichment of *Streptococcus* and deficiency in *Staphylococcus* were evidenced in lung cancer-associated microbiota (Liu et al., 2017).

The lung microbiome also affects the response of lung cancer cells to chemotherapy (Wang et al., 2021). Bacteria colonizing the TME can suppress immune response against tumor cells (Jin et al., 2019). Notably, IL17-producing γδ T cells are associated with the development of lung cancer. Moreover, the release of IL17 and IL23 can boost inflammation in lung tissues and favor the lung cancer cells proliferation (Jin et al., 2019). Antibiotic treatment can alleviate the tumor burden in the lung (Jin et al., 2019).

## 12 Conclusions and future perspectives

**Figure 5:**
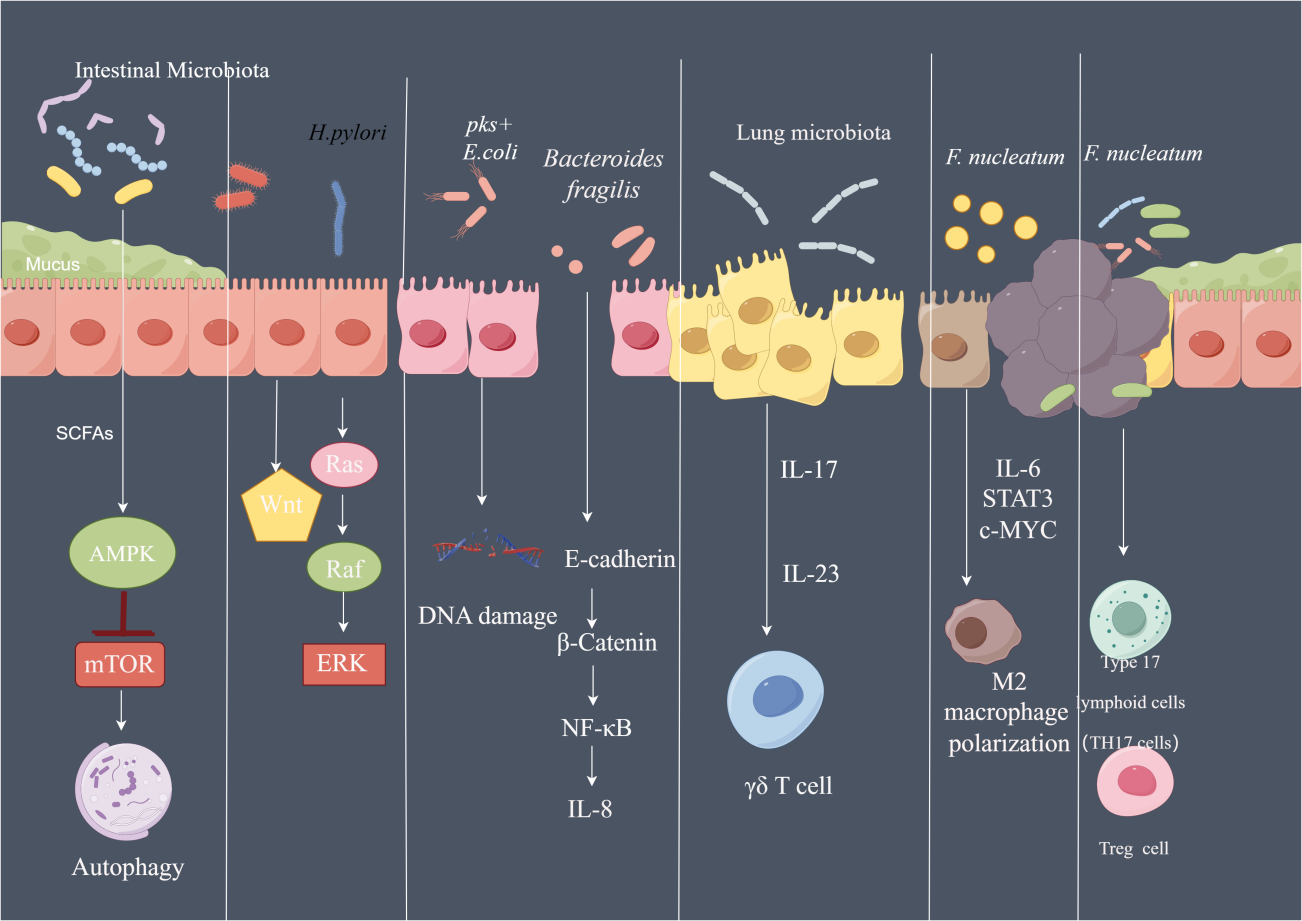
The pathways affected by microbiota in cancer SCFA of the microbiota can activate AMPK signaling pathway and inhibit mTOR signaling, further activate autophagy machinery. Phosphorylated CagA of *H. pylori* can activate Ras/Raf/ERK signaling and Wnt signaling in gastric epithelial cells. pks+ *E. coli* led to DNA damage of the intestinal epithelial cell. *Bacteroides fragilis* induce th production of IL-8 by activating E-cadherin/β-catenin/NF-κB signaling pathway. Lung microbiota promote the development of lung Cancer via γδ T Cells which can be activated by Myd88-dependent IL-1β and IL-23 induced by commensal bacteria. *F.nucleatum* regulate the polarization of macrophage to M2 phenotype by secreting IL-6, and activate IL-6/STAT3/c-MYC signaling. By Figdraw.

Microbiota promote tumor development, modulated the tumor environment to benefit cancer cells and effect the responses to chemotherapy (**Figure 5**). Comprehensive research is warranted to address numerous unanswered questions in the interaction of microbiota and host cells. Part of the bacteria play a driver function in the carcinogenesis, part of the bacteria plays the passenger function (Tjalsma et al., 2012). For example, *Salmonella* and *Citrobacter* can function as the driver of colon cancer carcinogenesis (Tjalsma et al., 2012), causing malignant transformation of the intestinal epithelial cell by metabolites (Avril and DePaolo, 2021). Therapeutic strategies targeting the microbiota is merging as a potential antitumor strategy. Investigation of the role of microbiota in cancer development may provide targets for anti-tumor therapy. Moreover, skewing of the microbiota balance may prevent tumor development. Finally, the microbiota composition in tumors may be used as an alternative biomarker for predicting prognosis and response to therapy.

## Author contributions

QL: Writing – original draft, Writing – review & editing.
